# Impact of Nutrient Starvation on Biofilm Formation in *Pseudomonas aeruginosa*: An Analysis of Growth, Adhesion, and Spatial Distribution

**DOI:** 10.3390/antibiotics13100987

**Published:** 2024-10-18

**Authors:** Laura Maria De Plano, Manuela Caratozzolo, Sabrina Conoci, Salvatore P. P. Guglielmino, Domenico Franco

**Affiliations:** 1 Department of Chemical, Biological, Pharmaceutical and Environmental Sciences (ChiBioFarAm), University of Messina, Viale F. Stagno d’Alcontres 31, 98166 Messina, Italy; 2 Department of Chemistry “Giacomo Ciamician”, Alma Mater Studiorum—University of Bologna, 40126 Bologna, Italy; 3 LAB Sense Beyond Nano—URT Department of Sciences Physics and Technologies of Matter (DSFTM) CNR, 98166 Messina, Italy

**Keywords:** bacterial adhesion, biofilm, *Pseudomonas aeruginosa*, nutrient starvation, fractal analysis

## Abstract

**Objectives:** This study investigates the impact of nutrient availability on the growth, adhesion, and biofilm formation of *Pseudomonas aeruginosa* ATCC 27853 under static conditions. **Methods:** Bacterial behaviour was evaluated in nutrient-rich Luria–Bertani (LB) broth and nutrient-limited M9 media, specifically lacking carbon (M9-C), nitrogen (M9-N), or phosphorus (M9-P). Bacterial adhesion was analysed microscopically during the transition from reversible to irreversible attachment (up to 120 min) and during biofilm production/maturation stages (up to 72 h). **Results:** Results demonstrated that LB and M9 media supported bacterial growth, whereas nutrient-starved conditions halted growth, with M9-C and M9-N inducing stationary phases and M9-P leading to cell death. Fractal analysis was employed to characterise the spatial distribution and complexity of bacterial adhesion patterns, revealing that nutrient-limited conditions affected both adhesion density and biofilm architecture, particularly in M9-C. In addition, live/dead staining confirmed a higher proportion of dead cells in M9-P over time (at 48 and 72 h). **Conclusions**: This study highlights how nutrient starvation influences biofilm formation and bacterial dispersion, offering insights into the survival strategies of *P. aeruginosa* in resource-limited environments. These findings should contribute to a better understanding of biofilm dynamics, with implications for managing biofilm-related infections and industrial biofouling.

## 1. Introduction

*Pseudomonas aeruginosa* (*P. aeruginosa*) is Gram-negative bacterium, particularly valuable for research among ESKAPE pathogens due to its exceptional adaptability, robust biofilm formation, and high antibiotic resistance [1,2,3]. It has a well-characterised quorum-sensing system that regulates virulence and an extensive genetic toolkit for functional studies [4,5,6]. Its versatility in adapting to different conditions, including those with frequent exposure to antibiotics or environmental stress, contributes to its involvement in 54% of deaths from infections [7]. In hospital settings, it is a notorious opportunistic human pathogen, causing acute and chronic infections in immunocompromised patients [8,9]. It is also the main cause of mortality due to chronic pulmonary infections in cystic fibrosis patients worldwide [10]. Its ability to form biofilm increases resistance to common antibiotics, such as β-lactams, fluoroquinolones, aminoglycosides, and polymyxins, making its eradication from infection sites very difficult [11,12]. Biomedical devices, such as catheters, heart valves, and prostheses, lead to chronic infection hardly being detected and health risks to patients [13,14,15,16]. Biofilm production is a common survival strategy for several bacteria to overcome adverse conditions [17]. In such conditions, bacteria convert their physiological state from free-floating (planktonic) to sessile cells, acquiring the ability to adhere, grow, and form biofilm on biotic or abiotic surfaces [18]. Within the biofilm, cells coordinates their metabolic activities, through quorum sensing (QS), regulating the expression of genes associated with different virulence factors [19,20]. The life cycle of a biofilm involves five main phases: reversible (i) and irreversible (ii) surface attachment, biofilm production (iii) and maturation (iv), and the dispersion (v) of single cells or EPS-included cell aggregates [21] (Figure S1). Surface attachment plays a key role for biofilm-associated bacteria, with weak attachments allowing bacteria to explore favourable sites for biofilm formation [22]. Bacterial motility systems (flagella and pili) and surface conditioning through polysaccharide secretion play a key role in this step [23,24,25]. Once a suitable surface is found, *P. aeruginosa* reduces its flagellar motility and switches to twitching motility (type IV pili), to move across the surface and release the exopolysaccharide Psl, enhancing surface attachment [26]. Surface characteristics are crucial in the initial adhesion phase, mainly due to hydrophobic and electrostatic interactions [27]. Positively charged surfaces generally favour adhesion due to the negative charge of the bacterial cell wall [28]. In Gram-negative bacteria like *P. aeruginosa*, lipopolysaccharides (LPSs) facilitate adhesion by imparting a strong negative charge to the bacterial surface. Abdel-Rhman suggests that LPSs in *P. aeruginosa* can promote and stabilise biofilm formation, also increasing their virulence [29,30]. During biofilm production, bacterial cells could be released, as microcolonies aggregates, to colonise new areas in response to environmental stimuli like nutrient scarcity to colonise new niches [31] in response to environmental stimuli, such as nutrient starvation [32]. *P. aeruginosa* under nutrient deprivation can trigger a series of stress responses, altering gene expression and metabolic pathways [33]. These responses can cause a shift from growth to maintenance, with some cells entering dormancy, while others prepare to disperse [34,35]. The dispersal mechanism may be affected by the limitation of specific nutrients. For instance, when carbon sources are depleted, *P. aeruginosa* may initiate the expression of dispersal-related genes, leading to the release of cells from the biofilm structure [32,36]. This process not only aids in nutrient acquisition by allowing bacteria to explore new environments but also facilitates the colonisation of new surfaces where conditions may be more favourable. Nutrient limitation can also reduce the extracellular polymeric substances (EPSs), weakening their structure and promoting regulated detachment. This controlled dispersal allows *P. aeruginosa* to adapt to its environment and relocate to areas with more abundant resources, enhancing survival in fluctuating conditions [37]. Each cell’s response within the microbial network shapes this global behaviour. For example, early adhesion can promote stable biofilm formation [38], while intercellular communication quickly alters gene expression, facilitating phenotypic adaptation in nutrient-limited environments [39,40,41]. The versatility in the adaptability and survival strategies of *P. aeruginosa* highlights the importance of understanding biofilm dynamics for the development of a control strategy for pathogenic infections and biofouling in various contexts.

The aim of this work is to investigate how nutrient availability affects the growth, adhesion, and biofilm formation of *P. aeruginosa* ATCC 27853. Its relevance to both clinical infections and environmental survival makes it an ideal model for studying bacterial pathogenesis, resistance mechanisms, and biofilm-related persistence, setting it apart from other ESKAPE pathogens. Specifically, this study aimed to examine bacterial behaviour in nutrient-rich and nutrient-limited media to understand how nutrient deprivation influences the transition from reversible to irreversible attachment and the subsequent development and maturation of biofilms.

## 2. Results

**Figure 1:**
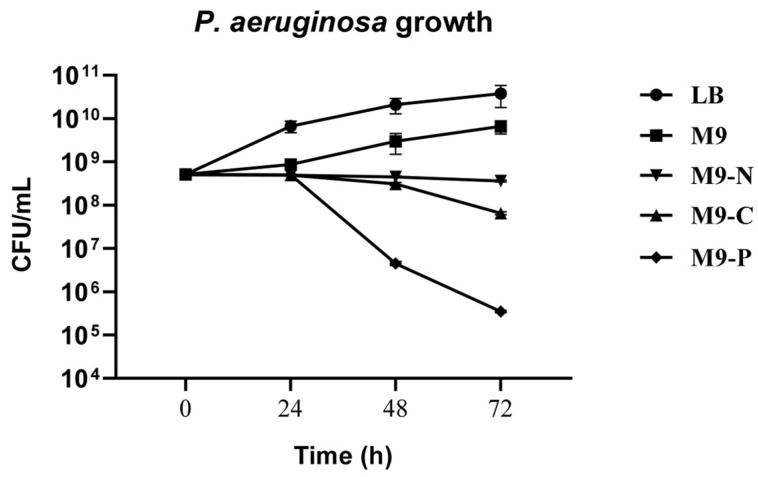
Viable cells (colony-forming units per mL, CFU/mL) of *P. aeruginosa* ATCC 27853 cultivated in Luria–Bertani (LB) broth, M9 medium with glucose as carbon source (M9) and under carbon (M9-C), nitrogen (M9-N) and phosphorus (M9-P) starvation, in static conditions at 37 °C (up to 72 h).

Media conditions used in this study included a nutrient-rich medium, Luria–Bertani (LB) broth, and nutrient-limited mineral media, specifically formulated from the minimal M9 medium. The LB medium represents a nutrient-rich environment that facilitates rapid bacterial growth, while the M9 medium, as a minimal medium with specific nutrients, allows for growth at a slower rate, highlighting the bacterium’s response to nutrient limitation. By including both media, we aim to show the baseline differences in bacterial growth under rich and minimal conditions, providing context for how nutrient-starved conditions further affect growth dynamics and biofilm formation. Nutrient-starved media included M9-C, M9-N, and M9-P, each lacking carbon (glucose), nitrogen (ammonia), and phosphorus (inorganic phosphate) sources, respectively. All culture conditions were evaluated for their influence on bacterial growth over a period of up to 3 days (Figure 1).

LB and M9 media permitted growth of *P. aeruginosa*, although with different growth rates. Otherwise, nutrient-starved conditions stopped *P. aeruginosa* growing, inducing cells to enter a stationary state (in the case of M9-C and M9-N) or death state (in the case of M9-P).

**Figure 2:**
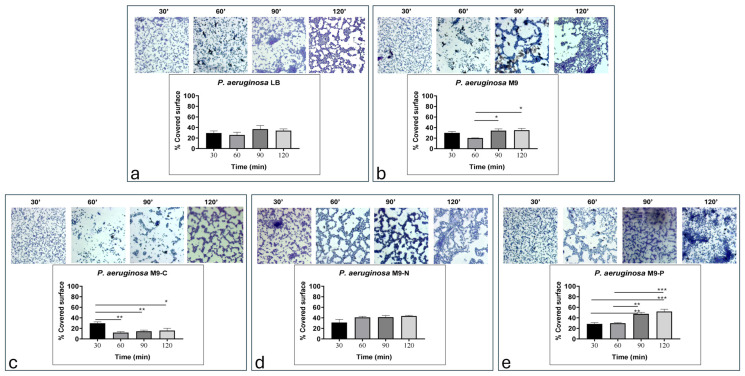
Representative images and quantitative evaluation (% of covered surface) of *P. aeruginosa* cells adhering in LB (**a**), M9 (**b**), M9-C (**c**), M9-N (**d**) and M9-P (**e**) media during transition stage from reversible to irreversible bacterial attachment (up to 120 min). Statistical significance of the difference between mean adhesion densities from each condition was evaluated using Tukey’s multiple comparison test and reported in Tables S1–S9. For ANOVA test, Tukey post hoc test for multiple comparison was used. One (*), two (**), and three (***) asterisks identify adjusted *p*-value < 0.01, 0.001, and 0.0001, respectively.

Figure 2 shows the adhesion pattern of *P. aeruginosa* cells adhering under different nutrient conditions during transition stage from reversible to irreversible bacterial attachment (up to 120 min).

No significant differences were observed in the surface coverage by bacteria for the tested conditions during the first 30 min of incubation (Table S1). At this time point, the bacteria appeared to completely cover the entire surface, except under the M9-N condition (Figure 2). In this case, it was already possible to observe the presence of gaps in different portions of the slide (Figure 2d). As incubation continued, circular gaps began to form in all conditions, with differences related to the specific nutrient limitations (Tables S2–S4).

Quantitatively, the LB condition (Figure 2a) showed a similar adhesion percentage during the entire observation period (29.4%, 25.8%, 36.9%, and 33.9% at 30, 60, 90, and 120 min, respectively). Although an increase in bacterial adhesion at 90 min was observed, no significant variations were detected compared to the other time points (Table S5). The M9 condition (Figure 2b) showed similar values to the LB condition (29.9%, 20.2%, 34.2%, and 35% at 30, 60, 90, and 120 min, respectively). However, at 60 min, adhesion density was significantly reduced compared to the later time points (Table S6). With regard to the M9-C condition (Figure 2c), results indicated the lowest adhesion densities (29.7%, 11.8%, 14.4%, and 15.9% at 30, 60, 90, and 120 min, respectively), with these differences becoming evident by 60 min of incubation (Table S7). The M9-N condition (Figure 2d) showed similar adhesion densities for the entire incubation period (31.2%, 40.7%, 41.1%, and 43.4% at 30, 60, 90, and 120 min, respectively). So, no significant differences were observed (Table S8). Finally, the M9-P condition (Figure 2e) appeared to significantly increase adhesion density only after 90 min of incubation (28.4%, 29.5%, 47.5%, and 51.2% at 30, 60, 90, and 120 min, respectively) (Table S9).

**Figure 3:**
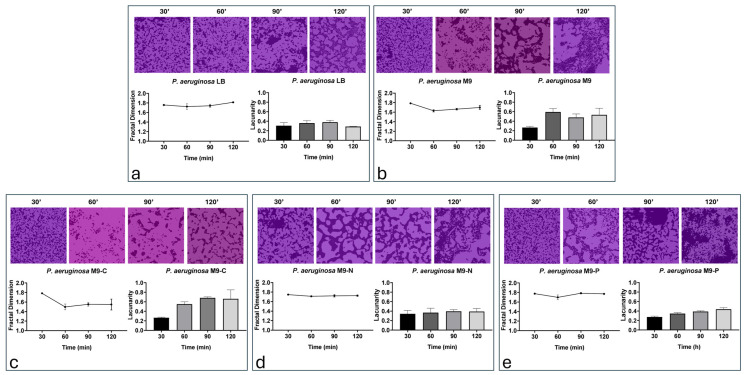
Fractal dimensions and lacunarity of *P. aeruginosa* cell adhering in LB (**a**), M9 (**b**), M9-C (**c**), M9-N (**d**) and M9-P (**e**) media during transition stage from reversible to irreversible bacterial attachment (up to 120 min). Background of representative images refers to the value in fractal dimension according to colour legend in Figure S2.

Fractal analysis was performed to provide more information about the pattern distribution of bacterial adhesion (Figure 3).

Analysis indicated a similar degree of complexity (fractal dimension between 1.65 and 1.8) of spatial distribution and self-similarity (lacunarity less than 0.65) in bacterial adhesion patterns across all nutritional conditions, except for the M9-C condition, which showed value under 1.6 and over 0.6 for fractal and self-similarity degree, respectively (Figure 3). With regard to LB and M9-N conditions, a linear fractal trend was observed during the monitored time points, while M9 and M9-P conditions showed an alteration downward of the values at 60 min, which was maintained in the later times points (Figure 3). Concerning the M9-C condition (Figure 3c), although the initial time point (30 min) showed typical values of a fractal spatial distribution, a decrease in fractal dimension and an increase in lacunarity were observed in the subsequent time points. These results suggest a reduction in the complexity and homogeneity of the bacterial adhesion pattern, likely driven by random and independent movements of individual cells within the bacterial population.

**Figure 4:**
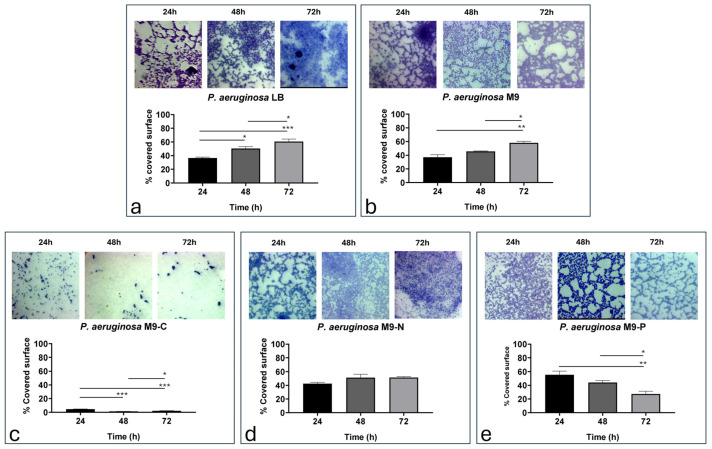
Representative images and quantitative evaluation (% of covered surface) of *P. aeruginosa* cells adhering in LB (**a**), M9 (**b**), M9-C (**c**), M9-N (**d**) and M9-P (**e**) media during biofilm production/maturation stage (up to 72 h). Statistical significance of the difference between mean adhesion densities from each condition was evaluated using Tukey’s multiple comparison test and reported in Tables S10–S19. For ANOVA test, Tukey post hoc test for multiple comparison was used. One (*), two (**), and three (***) asterisks identify adjusted *p*-value < 0.01, 0.001, and 0.0001, respectively.

Figure 4 shows the surface coverage of bacterial biofilm under different nutritional conditions over 72 h.

After 24 h of incubation, each condition exhibited a similar adhesion density to those recorded at 120 min, except for M9-C (Figure S3 and Tables S10–S14). In quantitative terms, we observe once again the lowest values for the M9-C condition.

The LB condition (Figure 4a) showed a significant increase in bacterial adhesion over the entire incubation period (36.5%, 50.5%, and 60.6% at 24, 48, and 72 h, respectively; Table S15). The M9 condition (Figure 4b) showed similar values to those of the LB condition (37.1%, 45.7%, and 58.1% at 24, 48, and 72 h, respectively) (Table S16). With regard to the M9-C condition (Figure 4c), results indicated the lowest bacterial adhesion densities (4.6%, 1.3%, and 2.2% at 24, 48, and 72 h, respectively), with resulting significant difference already demonstrated at 24 versus 48 h of incubation (Table S17). The M9-N condition (Figure 4d) showed similar values of adhesion for all time points evaluated in this study (42.4%, 51.2%, and 52.4% at 24, 48, and 72 h, respectively; Table S18). Finally, the M9-P condition (Figure 4e) appeared to significantly decrease the adhesion values during incubation times (55.3%, 44.2%, and 27.3% at 24, 48, and 72 h, respectively; Table S19).

**Figure 5:**
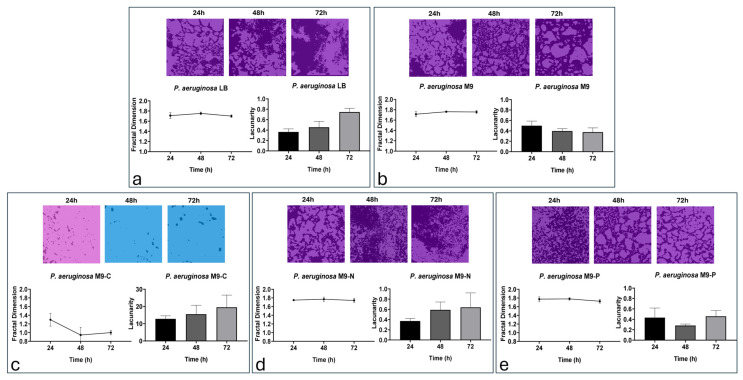
Fractal dimensions and lacunarity of *P. aeruginosa* cells adhering in LB (**a**), M9 (**b**), M9-C (**c**), M9-N (**d**) and M9-P (**e**) media during biofilm production/maturation stages (up to 72 h). Background of representative images refers to the value in fractal dimension value according to colour legend in Figure S2.

In this case, as well, we performed the fractal analysis to obtain information about the distribution pattern of cells within the biofilm structure (Figure 5).

All nutritional conditions indicated a similar degree of complexity (fractal dimension between 1.73 and 1.8) of spatial distribution and self-similarity (lacunarity less than 0.65) of bacterial adhesion patterns, except for the M9-C condition (Figure 5). The M9-C condition (Figure 5c) appeared to follow a random adhesion pattern, as confirmed by the variation in fractal dimensions at different time points and the high values for lacunarity (values above 10). In addition, fractal dimensions were maintained across varying magnification scale varies, further confirming the multi-level biofilm organisation pattern for tested conditions (Figure S4).

**Figure 6:**
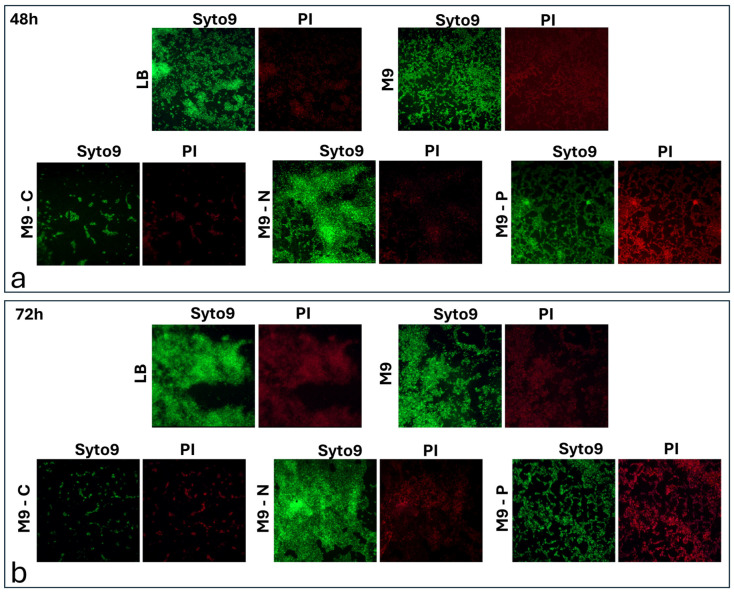
Live/dead staining of *P. aeruginosa* cell adhering under different nutrient conditions during biofilm production/maturation stage (48 (**a**) and 72 h (**b**)).

Live/dead staining at 48 and 72 h was performed to obtain information about the physiological states of adhering cells (Figure 6).

Fluorescent images showed a low level of dead cells for all nutritional conditions, except for the M9-P condition. Moreover, the distribution of the dead (red fluorescence) and live cells (green fluorescence) appeared similar, suggesting that the dead cells could serve as a substrate for the attachment of the new cells. The M9-P condition (Figure 6) indicated an increase in dead cells replacing live cells, mainly at 72 h. In this case, an increase in the size of the holes was also observed, confirming the drastic decrease in bacterial adhesion on the surface.

## 3. Discussion

In this work, we investigate the impact of nutrient availability on the growth, adhesion, and biofilm formation of *P. aeruginosa*. Specifically, we considered two main phases of biofilm production: (i) the transition stage from reversible to irreversible (within 2 h of incubation) and (ii) biofilm production/maturation (from 24 to 72 h). Since M9 is a defined minimal medium, it provides only the essential nutrients needed for growth, without additional energy sources or growth factors. According to that, we observed that LB promoted faster growth, underscoring the importance of a nutrient-rich environment, while in M9, *P. aeruginosa* grew more slowly, resulting in a longer lag phase and a lower exponential growth rate (Figure 1). On the other hand, cell adhesion was not significantly affected during the incubation periods being studied, with only a reduction in biofilm mass and structure resulting from live/dead staining (Figure 6). These findings were probably due to the lack of abundant nutrients that restricted the bacterium’s ability to produce extracellular polysaccharides and other biofilm components [42,43]. When the M9 minimal medium was deprived of a macronutrient (i.e., carbon, nitrogen, or phosphate), *P. aeruginosa* growth was stopped. Specifically, we found that carbon (M9-C) and nitrogen (M9-N) starvation halted growth, pushing cells into a stationary state, suggesting that *P. aeruginosa* adapts to nutrient-poor environments by limiting replication. In contrast, phosphorus limitation (M9-P) led to cell death, highlighting phosphorus’s critical role not only for growth but also for bacterial survival (Figure 1). Carbon starvation resulted as a critical condition for *P. aeruginosa* biofilms in all incubation periods, affecting the behaviour and survival of the bacteria. This resulted in the lowest adhesion density within the first hours of incubation, along with a reduction in the fractal dimension and an increase in lacunarity. Overall, these findings indicate that carbon is essential for robust initial and more organised bacterial attachment to the surface. This condition was maintained for all incubation periods and exhibited severely reduced biofilm formation, with low adhesion levels, indicating the cells’ inability to establish a stable, complex biofilm structure. Numerous studies associate the reduction in biofilm production with the triggering biofilm dispersal, a process where cells leave the biofilm and return to a planktonic (free-floating) state [32,44]. Similarly, our results showed a reduction in biofilm production associated with alterations in cell adhesion to the surface during the early stages of incubation. The lack of an alternative carbon source seems to limit biomass production, likely due to the arrest of cell growth and the inability to produce extracellular matrix components. On the other hand, some cells maintain their ability to adhere to the surface throughout the incubation period, suggesting that different survival strategies might be adopted within the bacterial population. Unlike in the M9-C condition, the M9-P condition significantly increased bacterial adhesion, with maximum biofilm production at 24 h. However, during later incubation periods, a progressive decrease in surface coverage was observed and correlated with an increase in cell death. These findings suggested that phosphorus limitation not only inhibits growth but also destabilises the biofilm structure over time. It is known that phosphate limitation alters the production of quorum-sensing signals in *P. aeruginosa* [45], affecting virulence and survival behaviours such as biofilm and swarming motility. The availability of phosphate plays a crucial role in the formation of biofilms by *P. aeruginosa*, primarily because phosphate is essential for the production of extracellular polysaccharides (EPSs), a key component of the biofilm matrix. The synthesis of these polysaccharides is regulated by several factors, including quorum-sensing and cyclic di-GMP levels, which influence the biofilm’s structural integrity and its resistance to environmental stresses [46]. Our results showed that under phosphate-starved conditions, *P. aeruginosa* would anticipate biofilm production, but it failed to maintain it over subsequent incubation periods. These behaviours may be explained because although phosphate starvation firstly aims to optimise the use of available phosphate [47,48], prolonged limitation may inhibit biofilm formation due to insufficient resources for building the biofilm matrix. Specifically, limiting phosphate availability could directly affect the production of extracellular polysaccharides and impose metabolic constraints, due to a reduction in energy production (through ATP synthesis). Overall, these findings suggest that while phosphate is a critical nutrient that can drive biofilm formation, its deficiency could also lead to a reduction in microbial growth and biofilm development. Finally, the M9-N condition in *P. aeruginosa* showed early adhesion comparable to that of complete medium. It is known that under nitrogen deficiency, *P. aeruginosa* tends to stop cellular growth, while increasing its survival metabolic activity, mainly related to bacterial dispersion and pathogenicity [49]. This response is strictly regulated by the alternative sigma factor RpoN that plays a role in nitrogen metabolism and the control of genes essential to virulence [50,51,52,53]. Under our experimental conditions, our findings suggest that nitrogen and carbon starvation led to the implementation of different survival strategies in *P. aeruginosa* without significant alteration in cell density.

## 4. Materials and Methods

**Bacterial strains and media.** In all experiments, *P. aeruginosa* ATCC 27853 (strain Boston 41501, isolated from blood culture) was used because it is well characterised and widely used in scientific research, ensuring consistency, reproducibility, and comparability with other studies. This allowed us to focus specifically on the impact of nutrient conditions on biofilm formation, generating a controlled and interpretable dataset. Bacterial glycerol stocks (culture/glycerol ratio 4:1), stored stably at −80 °C, were gradually thawed by one-hour steps at −20 °C, 4 °C, and room temperature (rt), then inoculated in 20 mL of Luria–Bertani broth (LB, Oxoid) contained in 100 mL Erlenmeyer’s flasks and incubated overnight at 37 °C with constant shaking at 250 rpm. Overnight cultures were harvested by centrifugation (6700× *g* for 10 min), washed twice with saline solution (0.9% NaCl), and suspended in the different media to obtain an optical density at 540 nm (OD_540_) of approximately 0.4 (Beckman (Brea, CA, USA) DU640 Spectrophotometer), corresponding to approximately 3–5 × 10^8^ colony-forming units (CFU) per mL. LB (composition per litre: Tryptone 10 g, NaCl 10 g, Yeast Extract 5 g) and M9 medium (composition per litre: 5.58 g Na_2_HPO_4_, 3.0 g KH_2_PO_4_, 0.5 g NaCl, 1 g NH_4_Cl, 10 mL 0.01 M CaCl_2_, 10 mL 0.1 M MgSO_4_, 1 mL 10 mM FeSO_4_, glucose 4 g). Carbon-limited medium (M9-C) was obtained depriving M9 medium of glucose. Nitrogen-limited medium (M9-N) was obtained replacing NH_4_Cl in the M9 medium with 1.17 g of NaCl. Phosphorus-limited medium (M9-P) was obtained replacing Na_2_HPO_4_ and KH_2_PO_4_ in the M9 medium with 2.8 g of NaCl and 1.64 g KCl, respectively, in order to maintain medium osmolarity. The pH was adjusted to 7.0 using 1 N NaOH, and media were sterilised by autoclaving at 121 °C for 20 min.

**Figure 7:**
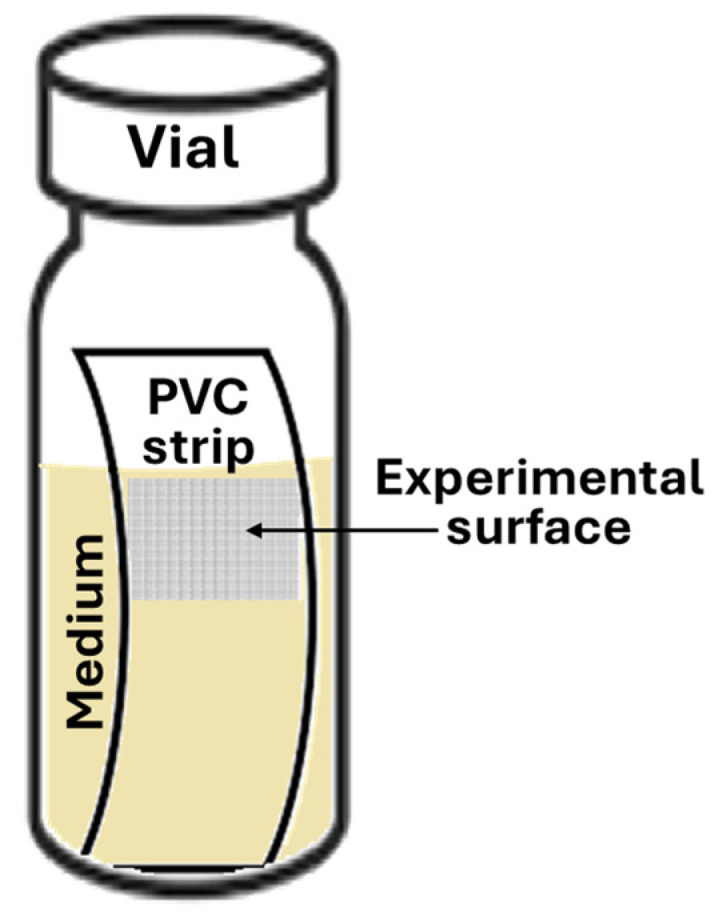
Schematic of bacterial growth chamber with vertical PVC strip. Arrow indicates surface’s next air–liquid interface considered for all experimental evaluations.

**Dynamics and kinetics of bacterial adhesion assay.** The dynamics and kinetics of bacterial adhesion were studied in bacterial growth chambers specifically designed to put polyvinylchloride (PVC) strips as abiotic surfaces in vertical position, in order to analyse adhesion by avoiding natural effects of sedimentation. The chambers consisted of glass autoclavable vials of 8.2 mm in diameter by 32 mm in length, with a bottleneck of 4.2 mm, closed by screwcaps with an air inlet. PVC strips (4 mm × 30 mm) were decontaminated with 70% ethanol, dried at room temperature, then inserted in the vials and kept in vertical position by the bottleneck. The chambers with PVC surfaces were filled with 0.8 mL of medium containing approximately 3–5 × 10^8^ CFU/mL, so that air–liquid interface was exactly at 14 mm from the bottom (about 1/2 of the height of PVC strip). The experimental setup is schematically shown in Figure 7.

Each cultural condition (in triplicate) was incubated at 37 °C in static conditions and evaluated at pre-set time intervals. Specifically, four time intervals (30, 60, 90, and 120 min) and three time (24, 48, and 72 h) intervals were considered for transition from reversible to irreversible bacterial attachment and production/maturation stage, respectively. At each time point, PVC strip was aseptically transferred into saline solution, washed gently thrice (avoiding drying), and stained for 15 min with Crystal Violet 0.1% *w*/*v* in in 95% ethanol at rt. After incubation periods, the excess dye was removed by washing twice with saline solution and samples dried under a laminar flow hood before microscopic observation. Optical images were observed by using a Leica (Wetzlar, Germany) DMRE microscope equipped with Leica Qwin software 2.7. Bacterial cell distribution of three different optical fields were acquired by a Leica DC300F Camera using a Leica C Plan 63× lens.

**Image analysis.** The processing and analysing of microscope images were carried out by using ImageJ (version 1.54g, National Institute of Health, Bethesda, MD, USA). Before being processed, colour image had to be converted to greyscale (8-bit) and adjusted threshold to highlight all cell structures from surface. This operation created a binary version of the original image with only two-pixel intensities (black = 0 and white = 255). Quantitative evaluation of cell adhesion was performed by using the analysis tool, in terms of integrated density (I.D. = N × (M − B), where N is the number of pixels in the selection, M is the average grey value of the pixels, and B is the most common pixel value. Box-counting fractal and lacunarity analysis was carried out by using FraLac plugin, keeping the original settings unchanged. In this case, for each processed image, analysis returned as output fractal dimension and lacunarity. Fractal dimension quantitatively measures structural properties and complexity of bacterial cell adhesion, providing information on how the cell distribution on the surface replicates itself at different scales of magnification. Structures with low fractal dimension (close to 1) were related to a simpler distribution, while those with high fractal dimension (closer to 2) to a more intricate and complex one. Lacunarity describes the degree of irregularity or “gaps” present in bacterial cell adhesion, quantifying the spatial distribution of voids (or gaps) within adherent cell mass and providing a measure of their heterogeneity. Fractal structure with low lacunarity were related to more uniform distribution of gaps and dense areas, whereas those with high lacunarity exhibits densely packed regions alternating with large empty spaces.

**Live/dead staining.** Bacterial adhesion and proliferation on the surface of PVC strips under different growing conditions were also assessed using a live/dead BacLight bacterial viability kit. In this case, the last time intervals (48 and 72 h) of biofilm production/maturation stage were considered. After each time point, PVC strip was aseptically transferred into sterile saline solution and washed gently thrice (avoiding drying). Then, each PVC strip was treated for 15 min with a mixture of SYTO^®^ 9 green-fluorescence nucleic acid stain and red-fluorescence dye (propidium iodide), so that bacteria with intact cellular membranes would be green-stained, whereas bacteria with damaged cellular membranes would be red-stained. After incubation periods, the excess dye was removed by washing twice with saline solution and the samples visualised under fluorescence microscopy using a Leica DMRE epifluorescence microscope with a Leica C Plan 63× lens, using a BP 515–560 nm excitation filter in combination with a LP 590 nm suppression filter.

**Statistical analysis.** All results were obtained from three independent experiments, expressed as means ± standard deviation and analysed by GraphPad software Prism 8. For ANOVA test, Tukey post hoc test for multiple comparison was used. One (*), two (**), and three (***) asterisks identify adjusted *p*-value < 0.01, 0.001, and 0.0001, respectively.

## 5. Conclusions

This study highlighted how nutrient availability significantly influences the growth, adhesion, and biofilm formation of *Pseudomonas aeruginosa* ATCC 27853, a bacterium known for its ability to colonise surfaces and form resilient biofilms. By using different culture media, including nutrient-rich Luria–Bertani (LB) broth and nutrient-limited M9 variants (M9-C, M9-N, M9-P), which are, respectively, lacking carbon, nitrogen, and phosphorus, this study explored bacterial behaviour under conditions of nutrient abundance and starvation. Our results suggest that *P. aeruginosa* can modulate its adhesion and growth based on nutrient availability, adapting to nutrient-poor conditions but revealing specific vulnerabilities under carbon and phosphorus starvation.

The finding that carbon starvation results in disorganised adhesion and reduced biofilm complexity highlights the crucial role of carbon not only as an energy source but also as a key factor regulating cell interactions. On the other hand, the significant reduction in cell viability under phosphorus starvation suggests that interventions targeting phosphorus availability could be an effective strategy to limit *P. aeruginosa* biofilm formation.

Overall, these findings could have important implications in both clinical and industrial settings for controlling *P. aeruginosa* infections and preventing biofouling. For this purpose, future studies should consider using flow cells or other dynamic culture systems to better simulate the dynamic environments that *P. aeruginosa* encounters in real-world settings. Additionally, investigating multiple strains from clinical or environmental isolates, which exhibit greater genetic diversity and phenotypic variability, would enhance our understanding of the bacterium’s behaviour under diverse conditions.

## Data Availability

Data are contained within this article or Supplementary Materials. The original contributions presented in this study are included in this article/Supplementary Materials, and further inquiries can be directed to the corresponding author/s. Dataset available on request from the authors The raw data supporting the conclusions of this article will be made available by the authors on request.

## Supplementary Materials

The following supporting information can be downloaded at https://www.mdpi.com/article/10.3390/antibiotics13100987/s1. Figure S1: Representation of life cycle of BF formation, from reversible adhesion of bacteria to biofilm dispersion. Figure S2: Legend background colours associated with the fractal dimension. Figure S3: Representative images and quantitative evaluation of *P. aeruginosa* cells adhering under different nutrient conditions at 2 h of incubation and during biofilm production/maturation stage (up to 72 h). Tukey’s multiple comparison tests for densities adhesion in each condition are reported in Tables S10–S19. Figure S4: Multilevel fractal analysis and adhesion patterns of *P. aeruginosa* cells adhering under different nutrient conditions during biofilm production/maturation stages. Table S1: Tukey’s multiple comparison test for adhesion density of *P. aeruginosa* after 30 min. Table S2: Tukey’s multiple comparison test for adhesion density of *P. aeruginosa* after 60 min. Table S3: Tukey’s multiple comparison test for adhesion density of *P. aeruginosa* after 90 min. Table S4: Tukey’s multiple comparison test for adhesion density of *P. aeruginosa* after 120 min. Table S5: Tukey’s multiple comparison test for adhesion density of *P. aeruginosa* in LB 30–120 min. Table S6: Tukey’s multiple comparison test for adhesion density of *P. aeruginosa* in M9 30–120 min. Table S7: Tukey’s multiple comparison test for adhesion density of *P. aeruginosa* in M9-C 30–120 min. Table S8: Tukey’s multiple comparison test for adhesion density of *P. aeruginosa* in M9-N 30–120 min. Table S9: Tukey’s multiple comparison test for adhesion density of *P. aeruginosa* in M9-P 30–120 min. Table S10: Tukey’s multiple comparison test for adhesion density of *P. aeruginosa* in LB 120 min–72 h. Table S11: Tukey’s multiple comparison test for adhesion density of *P. aeruginosa* in M9 120 min–72 h. Table S12: Tukey’s multiple comparison test for adhesion density of *P. aeruginosa* in M9-C 120 min–72 h. Table S13: Tukey’s multiple comparison test for adhesion density of *P. aeruginosa* in M9-N 120 min–72 h. Table S14: Tukey’s multiple comparison test for adhesion density of *P. aeruginosa* in M9p 120 min–72 h. Table S15: Tukey’s multiple comparison test for adhesion density of *P. aeruginosa* in LB 120 min–72 h. Table S16: Tukey’s multiple comparison test for adhesion density of *P. aeruginosa* in M9 120 min–72 h. Table S17: Tukey’s multiple comparison test for adhesion density of *P. aeruginosa* in M9-C. Table S18: Tukey’s multiple comparison test for adhesion density of *P. aeruginosa* in M9-N. Table S19: Tukey’s multiple comparison test for adhesion density of *P. aeruginosa* in M9-P.
