# Supplementary material for: Impact of Nutrient Starvation on Biofilm Formation in Pseudomonas aeruginosa: An Analysis of Growth, Adhesion, and Spatial Distribution
Source: Antibiotics (Basel). 2024 Oct 18;13(10):987. doi: 10.3390/antibiotics13100987 (PMC11504098; doi:10.3390/antibiotics13100987)
Supplement: Supplementary file 1 [file antibiotics-13-00987-s001.zip › antibiotics-3253059-supplementary.pdf]

Supporting Information

Impact of Nutrient Starvation on Biofilm Formation in Pseudomonas aeruginosa: An Analysis of Growth, Adhesion, and Spatial Distribution

Laura Maria De Plano [1], Manuela Caratozzolo [1], Sabrina Conoci [1,2,3,*], Salvatore P. P. Guglielmino [1] and Domenico Franco [1,*]

[1] Department of Chemical, Biological, Pharmaceutical and Environmental Sciences (ChiBioFarAm), University of Messina, Viale F. Stagno d'Alcontres 31, 98166 Messina, Italy;
[2] Department of Chemistry "Giacomo Ciamician", Alma Mater Studiorum—University of Bologna, 40126 Bologna, Italy
[3] LAB Sense Beyond Nano—URT Department of Sciences Physics and Technologies of Matter (DSFTM) CNR, 98166 Messina, Italy
* Correspondence: sabrina.conoci@unime.it (S.C.); domenico.franco@unime.it (D.F.)

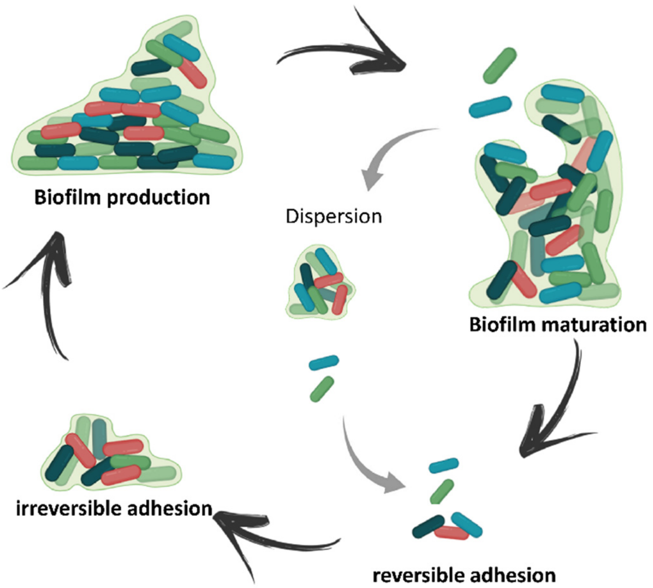

Figure S1. Representation of life cycle of BF formation, from reversible adhesion of bacteria to biofilm dispersion.

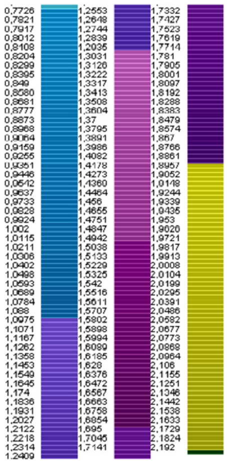

Figure S2. Legend background colours associated with the fractal dimension.

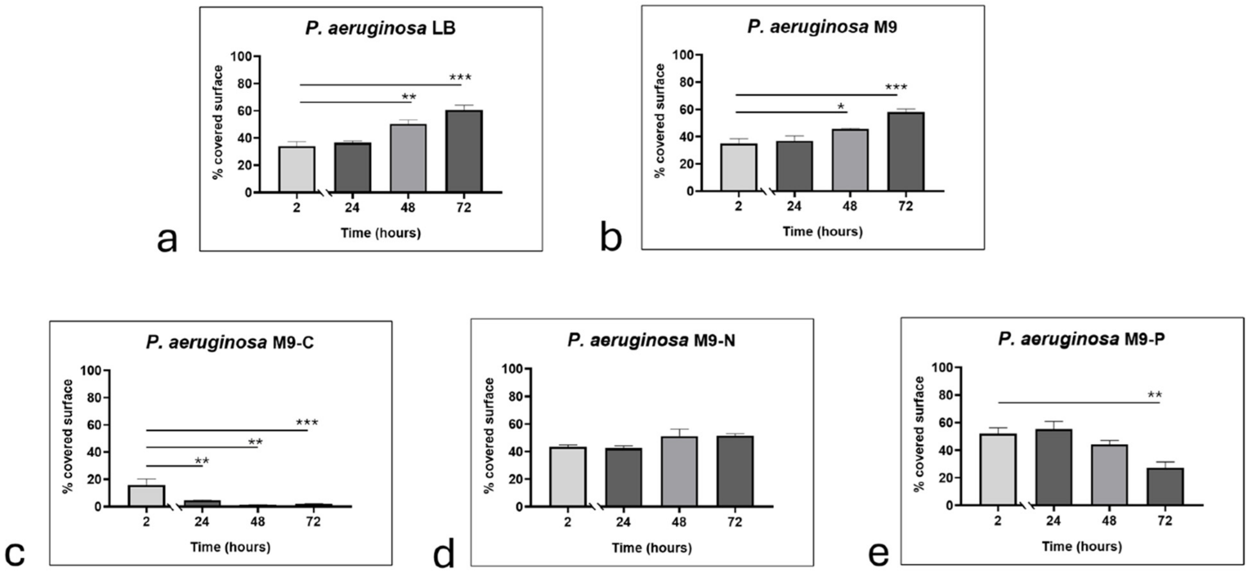

Figure S3. Representative images and quantitative evaluation of *P. aeruginosa* cells adhering under different nutrient conditions at 2 hour of incubation and during biofilm production/maturation stage (up to 72 hours). Statistical significance of the difference between means adhesion densities from each condition was evaluated by Tukey's multiple comparisons test and reported in Tables S10-S19.

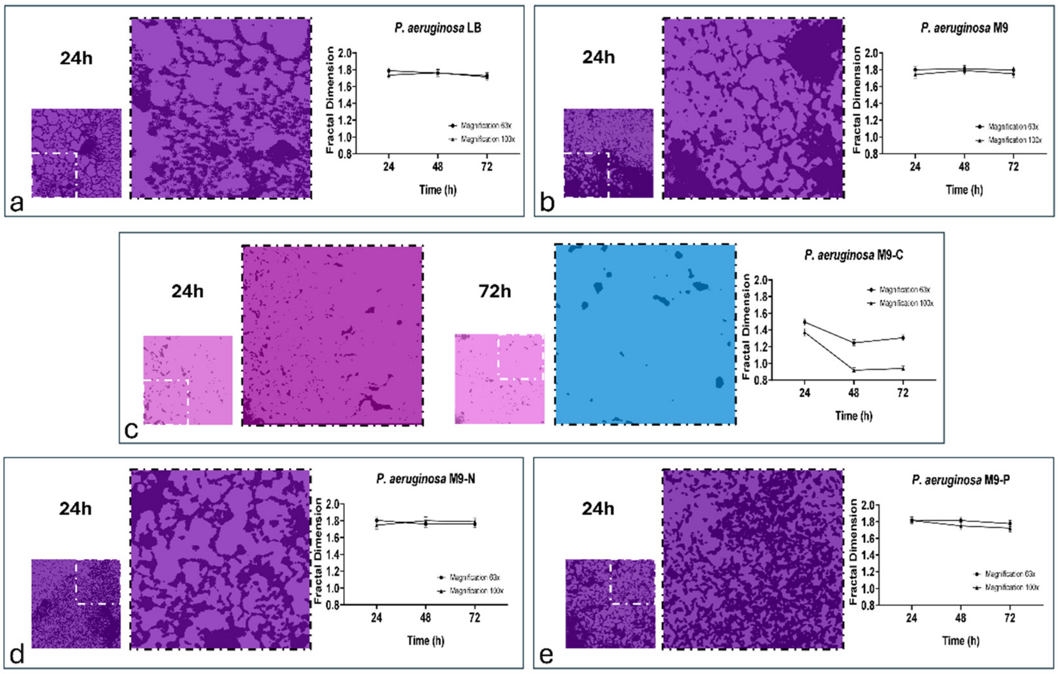

Figure S4. Multilevel fractal analysis and adhesion patterns of *P. aeruginosa* cells adhering under different nutrient conditions during biofilm production/maturation stages.

Table S1: Tukey's multiple comparisons test for adhesion density of *P. aeruginosa* after 30 minutes

Incubation time	Mean Diff,	95,00% CI of diff,	Significant?	Summary	Adjusted P Value
LB 30' vs. M9 30'	-0,4200	-13,96 to 13,12	No	Ns	>0,9999
LB 30' vs. M9-C 30'	-0,3033	-13,84 to 13,24	No	Ns	>0,9999
LB 30' vs. M9-N 30'	-1,727	-15,27 to 11,81	No	Ns	0,9790
LB 30' vs. M9-P 30'	1,053	-12,49 to 14,59	No	Ns	0,9967
M9 30' vs. M9-C 30'	0,1167	-13,42 to 13,66	No	Ns	>0,9999
M9 30' vs. M9-N 30'	-1,307	-14,85 to 12,23	No	Ns	0,9925
M9 30' vs. M9-P 30'	1,473	-12,07 to 15,01	No	Ns	0,9883
M9-C 30' vs. M9-N 30'	-1,423	-14,96 to 12,12	No	Ns	0,9897
M9-C 30' vs. M9-P 30'	1,357	-12,18 to 14,90	No	Ns	0,9914
M9-N 30' vs. M9-P 30'	2,780	-10,76 to 16,32	No	Ns	0,8939

One asterisk (*) identify adjusted P values between 0.01 and 0.001; two asterisks (**) identify adjusted P values between 0.001 and 0.0001; three asterisks (***) identify adjusted P values less than 0.0001

Table S2: Tukey's multiple comparisons test for adhesion density of *P. aeruginosa* after 60 minutes

Incubation time	Mean Diff,	95,00% CI of diff,	Significant?	Summary	Adjusted P Value
LB 60' vs. M9 60'	5,560	-4,258 to 15,38	No	Ns	0,1771
LB 60' vs. M9-C 60'	11,42	1,602 to 21,24	Yes	*	0,0036
LB 60' vs. M9-N 60'	-14,93	-24,75 to -5,116	Yes	**	0,0005
LB 60' vs. M9-P 60'	-3,707	-13,52 to 6,111	No	Ns	0,5081
M9 60' vs. M9-C 60'	5,860	-3,958 to 15,68	No	Ns	0,1459
M9 60' vs. M9-N 60'	-20,49	-30,31 to -10,68	Yes	***	<0,0001
M9 60' vs. M9-P 60'	-9,267	-19,08 to 0,5511	No	Ns	0,0144
M9-C 60' vs. M9-N 60'	-26,35	-36,17 to -16,54	Yes	***	<0,0001
M9-C 60' vs. M9-P 60'	-15,13	-24,94 to -5,309	Yes	**	0,0004
M9-N 60' vs. M9-P 60'	11,23	1,409 to 21,04	Yes	*	0,0040

One asterisk (*) identify adjusted P values between 0.01 and 0.001; two asterisks (**) identify adjusted P values between 0.001 and 0.0001; three asterisks (***) identify adjusted P values less than 0.0001

Table S3: Tukey's multiple comparisons test for adhesion density of *P. aeruginosa* after 90 minutes

Incubation time	Mean Diff,	95,00% CI of diff,	Significant?	Summary	Adjusted P Value
LB 90' vs. M9 90'	2,707	-11,62 to 17,04	No	ns	0,9187
LB 90' vs. M9-C 90'	25,01	10,68 to 39,34	Yes	**	0,0001
LB 90' vs. M9-N 90'	-4,237	-18,57 to 10,09	No	Ns	0,7069
LB 90' vs. M9-P 90'	-10,65	-24,98 to 3,678	No	Ns	0,0554
M9 90' vs. M9-C 90'	22,30	7,972 to 36,63	Yes	**	0,0004
M9 90' vs. M9-N 90'	-6,943	-21,27 to 7,388	No	Ns	0,2903
M9 90' vs. M9-P 90'	-13,36	-27,69 to 0,9716	No	Ns	0,0156
M9-C 90' vs. M9-N 90'	-29,25	-43,58 to -14,92	Yes	***	<0,0001
M9-C 90' vs. M9-P 90'	-35,66	-49,99 to -21,33	Yes	***	<0,0001
M9-N 90' vs. M9-P 90'	-6,417	-20,75 to 7,915	No	Ns	0,3566

One asterisk (*) identify adjusted P values between 0.01 and 0.001; two asterisks (**) identify adjusted P values between 0.001 and 0.0001; three asterisks (***) identify adjusted P values less than 0.0001

Table S4: Tukey's multiple comparisons test for adhesion density of *P. aeruginosa* after 120 minutes

Incubation time	Mean Diff,	95,00% CI of diff,	Significant?	Summary	Adjusted P Value
LB 120' vs. M9 120'	-1,020	-13,64 to 11,60	No	Ns	0,9962
LB 120' vs. M9-C120'	18,04	5,420 to 30,67	Yes	**	0,0007
LB 120' vs. M9-N 120'	-9,497	-22,12 to 3,127	No	Ns	0,0521
LB 120' vs. M9-P 120'	-18,01	-30,63 to -5,383	Yes	**	0,0008
M9 120' vs. M9-C120'	19,06	6,440 to 31,69	Yes	**	0,0005
M9 120' vs. M9-N 120'	-8,477	-21,10 to 4,147	No	Ns	0,0895
M9 120' vs. M9-P 120'	-16,99	-29,61 to -4,363	Yes	*	0,0012
M9-C120' vs. M9-N 120'	-27,54	-40,16 to -14,92	Yes	***	<0,0001
M9-C120' vs. M9-P 120'	-36,05	-48,67 to -23,43	Yes	***	<0,0001
M9-N 120' vs. M9-P 120'	-8,510	-21,13 to 4,114	No	Ns	0,0880

One asterisk (*) identify adjusted P values between 0.01 and 0.001; two asterisks (**) identify adjusted P values between 0.001 and 0.0001; three asterisks (***) identify adjusted P values less than 0.0001

Table S5: Tukey's multiple comparisons test for adhesion density of *P. aeruginosa* in LB 30-120 minutes

Incubation time	Mean Diff,	95,00% CI of diff,	Significant?	Summary	Adjusted P Value
30 vs. 60	3,633	-14,54 to 21,80	No	Ns	0,8166
30 vs. 90	-7,427	-25,60 to 10,74	No	Ns	0,3426
30 vs. 120	-4,500	-22,67 to 13,67	No	Ns	0,7069
60 vs. 90	-11,06	-29,23 to 7,110	No	Ns	0,1059
60 vs. 120	-8,133	-26,30 to 10,04	No	Ns	0,2767
90 vs. 120	2,927	-15,24 to 21,10	No	Ns	0,8917

One asterisk (*) identify adjusted P values between 0.01 and 0.001; two asterisks (**) identify adjusted P values between 0.001 and 0.0001; three asterisks (***) identify adjusted P values less than 0.0001

Table S6: Tukey's multiple comparisons test for adhesion density of *P. aeruginosa* in M9 30-120 minutes

Incubation time	Mean Diff,	95,00% CI of diff,	Significant?	Summary	Adjusted P Value
30 vs. 60	9,613	-0,2715 to 19,50	No	Ns	0,0117
30 vs. 90	-4,300	-14,18 to 5,585	No	Ns	0,2969
30 vs. 120	-5,100	-14,98 to 4,785	No	Ns	0,1862
60 vs. 90	-13,91	-23,80 to -4,028	Yes	*	0,0012
60 vs. 120	-14,71	-24,60 to -4,828	Yes	**	0,0008
90 vs. 120	-0,8000	-10,68 to 9,085	No	Ns	0,9836

One asterisk (*) identify adjusted P values between 0.01 and 0.001; two asterisks (**) identify adjusted P values between 0.001 and 0.0001; three asterisks (***) identify adjusted P values less than 0.0001

Table S7: Tukey's multiple comparisons test for adhesion density of *P. aeruginosa* in M9-C 30-120 minutes

Incubation time	Mean Diff,	95,00% CI of diff,	Significant?	Summary	Adjusted P Value
30 vs. 60	17,89	7,478 to 28,30	Yes	**	0,0003
30 vs. 90	15,36	4,948 to 25,77	Yes	**	0,0009
30 vs. 120	13,85	3,438 to 24,26	Yes	*	0,0017
60 vs. 90	-2,530	-12,94 to 7,879	No	Ns	0,7180
60 vs. 120	-4,040	-14,45 to 6,369	No	Ns	0,3819
90 vs. 120	-1,510	-11,92 to 8,899	No	Ns	0,9173

One asterisk (*) identify adjusted P values between 0.01 and 0.001; two asterisks (**) identify adjusted P values between 0.001 and 0.0001; three asterisks (***) identify adjusted P values less than 0.0001

Table S8: Tukey's multiple comparisons test for adhesion density of *P. aeruginosa* in M9-N 30-120 minutes

Incubation time	Mean Diff,	95,00% CI of diff,	Significant?	Summary	Adjusted P Value
30 vs. 60	-9,573	-22,78 to 3,631	No	Ns	0,0516
30 vs. 90	-9,937	-23,14 to 3,268	No	Ns	0,0435
30 vs. 120	-12,27	-25,47 to 0,9348	No	Ns	0,0150
60 vs. 90	-0,3633	-13,57 to 12,84	No	Ns	0,9993
60 vs. 120	-2,697	-15,90 to 10,51	No	Ns	0,8074
90 vs. 120	-2,333	-15,54 to 10,87	No	Ns	0,8636

One asterisk (*) identify adjusted P values between 0.01 and 0.001; two asterisks (**) identify adjusted P values between 0.001 and 0.0001; three asterisks (***) identify adjusted P values less than 0.0001

Table S9: Tukey's multiple comparisons test for adhesion density of *P. aeruginosa* in M9-P 30-120 minutes

Incubation time	Mean Diff,	95,00% CI of diff,	Significant?	Summary	Adjusted P Value
30 vs. 60	-1,127	-11,75 to 9,497	No	Ns	0,9646
30 vs. 90	-19,13	-29,76 to -8,510	Yes	**	0,0002
30 vs. 120	-23,56	-34,18 to -12,94	Yes	***	<0,0001
60 vs. 90	-18,01	-28,63 to -7,383	Yes	**	0,0003
60 vs. 120	-22,43	-33,06 to -11,81	Yes	***	<0,0001
90 vs. 120	-4,427	-15,05 to 6,197	No	Ns	0,3283

One asterisk (*) identify adjusted P values between 0.01 and 0.001; two asterisks (**) identify adjusted P values between 0.001 and 0.0001; three asterisks (***) identify adjusted P values less than 0.0001

Table S10: Tukey's multiple comparisons test for adhesion density of *P. aeruginosa* in LB 120min-72hs

Incubation time	Mean Diff,	95,00% CI of diff,	Significant?	Summary	Adjusted P Value
120 min vs. 24hs	-2,553	-13,08 to 7,976	No	Ns	0,7194
120 min vs. 48hs	-16,54	-27,07 to -6,014	Yes	**	0,0006
120 min vs. 72hs	-26,69	-37,22 to -16,16	Yes	***	<0,0001
24hs vs. 48hs	-13,99	-24,52 to -3,461	Yes	*	0,0018
24hs vs. 72hs	-24,13	-34,66 to -13,60	Yes	***	<0,0001
48hs vs. 72hs	-10,14	-20,67 to 0,3860	No	NS	0,0123

One asterisk (*) identify adjusted P values between 0.01 and 0.001; two asterisks (**) identify adjusted P values between 0.001 and 0.0001; three asterisks (***) identify adjusted P values less than 0.0001

Table S11: Tukey's multiple comparisons test for adhesion density of *P. aeruginosa* in M9 120min-72hs

Incubation time	Mean Diff,	95,00% CI of diff,	Significant?	Summary	Adjusted P Value
120 min vs. 24hs	-2,130	-11,89 to 7,627	No	Ns	0,7761
120 min vs. 48hs	-10,79	-20,55 to -1,037	Yes	*	0,0055
120 min vs. 72hs	-23,19	-32,94 to -13,43	Yes	***	<0,0001
24hs vs. 48hs	-8,663	-18,42 to 1,093	No	Ns	0,0192
24hs vs. 72hs	-21,06	-30,81 to -11,30	Yes	***	<0,0001
48hs vs. 72hs	-12,39	-22,15 to -2,637	Yes	*	0,0023

One asterisk (*) identify adjusted P values between 0.01 and 0.001; two asterisks (**) identify adjusted P values between 0.001 and 0.0001; three asterisks (***) identify adjusted P values less than 0.0001

Table S12: Tukey's multiple comparisons test for adhesion density of *P. aeruginosa* in M9-C 120min-72hs

Incubation time	Mean Diff,	95,00% CI of diff,	Significant?	Summary	Adjusted P Value
120 min vs. 24hs	11,32	3,620 to 19,02	Yes	**	0,0009
120 min vs. 48hs	14,57	6,870 to 22,27	Yes	**	0,0002
120 min vs. 72hs	13,67	5,970 to 21,37	Yes	**	0,0002
24hs vs. 48hs	3,250	-4,450 to 10,95	No	Ns	0,3188
24hs vs. 72hs	2,350	-5,350 to 10,05	No	Ns	0,5663
48hs vs. 72hs	-0,9000	-8,600 to 6,800	No	Ns	0,9537

One asterisk (*) identify adjusted P values between 0.01 and 0.001; two asterisks (**) identify adjusted P values between 0.001 and 0.0001; three asterisks (***) identify adjusted P values less than 0.0001

Table S13: Tukey's multiple comparisons test for adhesion density of *P. aeruginosa* in M9-N 120min-72hs

Incubation time	Mean Diff,	95,00% CI of diff,	Significant?	Summary	Adjusted P Value
120 min vs. 24hs	1,053	-9,122 to 11,23	No	Ns	0,9669
120 min vs. 48hs	-7,813	-17,99 to 2,362	No	Ns	0,0396
120 min vs. 72hs	-8,020	-18,20 to 2,155	No	Ns	0,0350
24hs vs. 48hs	-8,867	-19,04 to 1,309	No	Ns	0,0212
24hs vs. 72hs	-9,073	-19,25 to 1,102	No	Ns	0,0188
48hs vs. 72hs	-0,2067	-10,38 to 9,969	No	Ns	0,9997

One asterisk (*) identify adjusted P values between 0.01 and 0.001; two asterisks (**) identify adjusted P values between 0.001 and 0.0001; three asterisks (***) identify adjusted P values less than 0.0001

Table S14: Tukey's multiple comparisons test for adhesion density of *P. aeruginosa* in M9-P 120min-72hs

Incubation time	Mean Diff,	95,00% CI of diff,	Significant?	Summary	Adjusted P Value
120 min vs. 24hs	-3,427	-18,89 to 12,03	No	ns	0,7685
120 min vs. 48hs	7,713	-7,748 to 23,17	No	ns	0,2060
120 min vs. 72hs	24,64	9,182 to 40,10	Yes	***	0,0005
24hs vs. 48hs	11,14	-4,322 to 26,60	No	ns	0,0530
24hs vs. 72hs	28,07	12,61 to 43,53	Yes	***	0,0002
48hs vs. 72hs	16,93	1,468 to 32,39	Yes	**	0,0059

One asterisk (*) identify adjusted P values between 0.01 and 0.001; two asterisks (**) identify adjusted P values between 0.001 and 0.0001; three asterisks (***) identify adjusted P values less than 0.0001

Table S15: Tukey's multiple comparisons test for adhesion density of *P. aeruginosa* in LB 24-72hs

Incubation time	Mean Diff,	95,00% CI of diff,	Significant?	Summary	Adjusted P Value
24 vs. 48	-13,99	-24,01 to -3,966	Yes	*	0,0019
24 vs. 72	-24,13	-34,16 to -14,11	Yes	***	<0,0001
48 vs. 72	-10,14	-20,17 to -0,1191	Yes	*	0,0095

One asterisk (*) identify adjusted P values between 0.01 and 0.001; two asterisks (**) identify adjusted P values between 0.001 and 0.0001; three asterisks (***) identify adjusted P values less than 0.0001

Table S16: Tukey's multiple comparisons test for adhesion density of *P. aeruginosa* in M9 24-72hs

Incubation time	Mean Diff,	95,00% CI of diff,	Significant?	Summary	Adjusted P Value
24 vs. 48	-8,663	-17,59 to 0,2613	No	Ns	0,0115
24 vs. 72	-21,06	-29,98 to -12,13	Yes	**	0,0001
48 vs. 72	-12,39	-21,32 to -3,469	Yes	*	0,0019

One asterisk (*) identify adjusted P values between 0.01 and 0.001; two asterisks (**) identify adjusted P values between 0.001 and 0.0001; three asterisks (***) identify adjusted P values less than 0.0001

Table S17: Tukey's multiple comparisons test for adhesion density of *P. aeruginosa* in M9-C 24-72hs

Incubation time	Mean Diff,	95,00% CI of diff,	Significant?	Summary	Adjusted P Value
24 vs. 48	3,250	2,485 to 4,015	Yes	***	<0,0001
24 vs. 72	2,350	1,585 to 3,115	Yes	***	<0,0001
48 vs. 72	-0,9000	-1,665 to -0,1354	Yes	*	0,0045

One asterisk (*) identify adjusted P values between 0.01 and 0.001; two asterisks (**) identify adjusted P values between 0.001 and 0.0001; three asterisks (***) identify adjusted P values less than 0.0001

Table S18: Tukey's multiple comparisons test for adhesion density of *P. aeruginosa* in M9-N 24-72hs

Incubation time	Mean Diff,	95,00% CI of diff,	Significant?	Summary	Adjusted P Value
24 vs. 48	-8,867	-20,45 to 2,717	No	Ns	0,0324
24 vs. 72	-9,073	-20,66 to 2,511	No	Ns	0,0295
48 vs. 72	-0,2067	-11,79 to 11,38	No	Ns	0,9965

One asterisk (*) identify adjusted P values between 0.01 and 0.001; two asterisks (**) identify adjusted P values between 0.001 and 0.0001; three asterisks (***) identify adjusted P values less than 0.0001

Table S19: Tukey's multiple comparisons test for adhesion density of *P. aeruginosa* in M9-P 24-72hs

Incubation time	Mean Diff,	95,00% CI of diff,	Significant?	Summary	Adjusted P Value
M9-P condition					
24 vs. 48	11,14	-4,559 to 26,84	No	Ns	0,0438
24 vs. 72	28,07	12,37 to 43,77	Yes	**	0,0005
48 vs. 72	16,93	1,231 to 32,63	Yes	*	0,0070

One asterisk (*) identify adjusted P values between 0.01 and 0.001; two asterisks (**) identify adjusted P values between 0.001 and 0.0001; three asterisks (***) identify adjusted P values less than 0.0001
